# Induction of a B-cell-dependent chronic arthritis with glucose-6-phosphate isomerase

**DOI:** 10.1186/ar1829

**Published:** 2005-09-20

**Authors:** Robert Bockermann, David Schubert, Thomas Kamradt, Rikard Holmdahl

**Affiliations:** 1 Section for Medical Inflammation Research, University of Lund, Lund, Sweden; 2 Deutsches Rheumaforschungszentrum Berlin, Berlin, Germany; 3 Deutsches Rheumaforschungszentrum Berlin, and Institut für Immunologie, Klinikum der FSU, Jena, Germany; 4 Section for Medical Inflammation Research, University of Lund, Lund, Sweden

## Abstract

Antibodies specific for glucose-6-phosphate isomerase (G6PI) from T-cell receptor transgenic K/BxN mice are known to induce arthritis in mice, and immunization of DBA/1 mice with G6PI led to acute arthritis without permanent deformation of their joints. Because rheumatoid arthritis is a chronic disease, we set out to identify the capacity of G6PI to induce chronic arthritis in mice. Immunization with recombinant human G6PI induced a chronically active arthritis in mice with a C3H genomic background, whereas the DBA/1 background allowed only acute arthritis and the C57BL/10 background permitted no or very mild arthritis. The disease was associated with the major histocompatibility region sharing an allelic association similar to that of collagen-induced arthritis (i.e. q > p > r). All strains developed a strong antibody response to G6PI that correlated only in the C3H.NB strain with arthritis severity. Similarly, a weak response to type II collagen in a few mice was observed, which was associated with arthritis in C3H.NB mice. Mice on the C3H background also developed ankylosing spondylitis in the vertebrae of the tail. Both C3H.Q and B10.Q mice deficient for B cells were resistant to arthritis. We conclude that G6PI has the ability to induce a chronic arthritis, which is MHC associated and B-cell dependent. Thus, there are striking similarities between this and the collagen-induced arthritis model.

## Introduction

Glucose-6-phosphate isomerase (G6PI) is a widely expressed protein with multiple functions. It is an essential cytosolic enzyme in the energy cycle and has glycolytic activity, but it also has additional functions as an extracellular signalling molecule. Thus, G6PI is also known as AMF (autocrine motility factor) and neuroleukine, and may play roles in both cancer and autoimmunity [1,2].

Coincidentally, it was found that G6PI plays an essential role in the development of arthritis in mice. This originally stemmed from the observation that a bovine pancreas ribonuclease specific T-cell receptor transgenic mouse crossed with NOD mice (the so-called K/BxN mouse) spontaneously developed arthritis. Through a series of elegant experiments it was demonstrated that this transgenic T-cell receptor recognized G6PI within the context of major histocompatibility complex (MHC) class II molecule A^g7 ^[3,4]. The transgenic autoreactive T cells triggered autoreactive B cells to produce arthritogenic antibodies specific to G6PI [4-6]. After transferring G6PI-reactive serum from arthritic K/BxN mice, these antibodies bound to peripheral joints and induced arthritis in a manner strikingly similar to that shown previously for antibodies to the cartilage-specific antigen collagen type II (CII) [7,8]. B-cell activation in response to G6PI appeared to occur primarily in lymph nodes draining the joints [9], indicating that recognition of G6PI is joint specific. The reason for this specificity is not apparent because the G6PI protein is a ubiquitous protein.

Even though there are inconsistent data regarding the role of G6PI in rheumatoid arthritis (RA) [10-13], it appears that antibodies to G6PI occur predominantly in patients with Felty's syndrome – a variant of RA [14]. It is still unclear whether this is a unique phenomenon of Felty's syndrome or whether it reflects a generalized higher autoreactivity in these patients. Support for the latter comes from a study using affinity purified sera from arthritis patients that compared different kinds of arthritides and suggested a role for G6PI antibodies [15]. G6PI is nevertheless an interesting autoantigen and may represent a unique pathway leading to aggressive subtypes of RA. It is thus interesting to investigate this model further and to compare it with other models, such as the classic collagen-induced arthritis (CIA) model [16]. Here we show that immunization with G6PI leads to a chronically active arthritis in mice with genes from the C3H background, and that susceptibility is both controlled by the MHC region and dependent on B cells.

## Materials and methods

### Mice

The mouse strains were bred and used in the animal facility of the Section for Medical Inflammation Research (University of Lund, Lund, Sweden). Mice of DBA/1J and C3H.NB origins were from Jackson Laboratories (Bar Harbor, Maine, USA), and those of B10.Q, B10.P and B10.RIII origins were from Dr Jan Klein (Max-Planck-Institut für Biologie, Abteilung Immungenetik, Tübingen, Germany). C3H.Q mice were established through backcrossing (4n) of the H2^q ^fragment derived from an original C3H.Q mouse into C3H.NB [17]. The human DR4 transgenic and the backcrossed B-cell deficient mouse strains were described previously [18-20].

Experimental mice were matched for sex and age in all experiments. The founder μMT mouse was kindly provided by Dr Werner Müller (Institute of Genetics, Cologne, Germany), which we backcrossed to B10.Q for 13 generations before they were intercrossed for the experiment. C3H.Q μMT mice were backcrossed for 10 generations and finally intercrossed.

The experiments were conducted in accordance with guidelines from the Swedish Ethical Committee.

### Antigens

Recombinant human G6PI was produced as previously described [21]. G6PI cDNA fragments were introduced into a modified pQE100 expression vector for expression of His-tagged proteins in *Escherichia coli *strain Bl21. Supernatants of bacterial lysates were subjected to purification over a Ni-NTA column (Qiagen, Hilden, Germany), in accordance with the manufacturer's instructions. Using the same strategy, human creatine kinase (hCK) and mouse G6PI (mG6PI) was produced. The purity of proteins was checked using standard SDS gels. Rat collagen type II (rCII) was prepared from SWARM chondrosarcoma by pepsin digestion [22] and further purified as described previously [23].

### Immunization and arthritis scoring

An emulsion for immunization was made by sonication, using an aliquot of human G6PI and complete Freund's adjuvant (Difco, Detroit, MI, USA), resulting in an emulsion of 2 mg/ml human G6PI. A total of 100 μl of the emulsion (200 μg G6PI) was injected at the base of the tail in each mouse. For the titration experiments G6PI was diluted with phosphate-buffered saline to adjust to the required concentration. In some experiments mice were given an intradermal boost with 50 μg G6PI in incomplete Freund's adjuvant. Mice were visually scored for arthritis using an extended scoring protocol ranging from 1 to 15 for each paw, allowing a maximum score of 60 per mouse. Each arthritic (red and swollen) toe and knuckle was scored as 1, whereas an affected ankle was scored as 5 (total: 15/paw) [24].

### Antibody analysis

Serum for analysis of antibody levels was taken at indicated time points and at the end of all experiments. Serum was diluted 1:1,000 for G6PI, mG6PI, hCK and 1:100 for rCII antibody analysis. ELISA Maxisorp plates (Nunc, Roskilde Denmark) were coated with 50 μl of 10 μg/ml of the recombinant proteins or rat CII. The amounts of total specific IgG was determined through quantitative ELISA using peroxidase-conjugated goat anti-mouse IgG (H+L; 115-035-062; Jackson ImmunoResearch, West Grove, PA, USA) secondary antibodies [25]. ABTS (2,2'-Azino-bis(3-Ethylbenzthiazoline-6-Sulfonic Acid), # 11204521001; Roche Diagnostics GmbH, Penzberg, Germany) was used as substrate. Values were measured at 405 nm and are expressed as optical density values.

### Histology

At the end of the experiments paws, knees, and tails were fixated in 4% paraformaldehyde for 24 hours and decalcified with EDTA. The paraffin sections were stained with haematoxylin and erythrosine [26].

### Statistical analysis

Frequency of arthritis was analyzed using the χ^2 ^test, and antibody levels and arthritis severity were analyzed using the Mann-Whitney U-test. Disease score and antibody correlations were analyzed using the Spearman rho correlation test from the StatView software package (Version 5.0.1, SAS Institute Inc., Cary, NC, USA).

## Results

### Titration of the arthritogenic dose of G6PI

DBA/1 mice were used to confirm induction of arthritis using human recombinant G6PI in our animal house and to titrate the dose. Almost 100% of the mice developed arthritis upon immunization with all of the doses used, although the severity was dose dependent. With the lowest dose (100 μg) the arthritis started as early as day 9 and subsequently progressed to a severe arthritis peaking 2 weeks after immunization.

**Figure 1:**
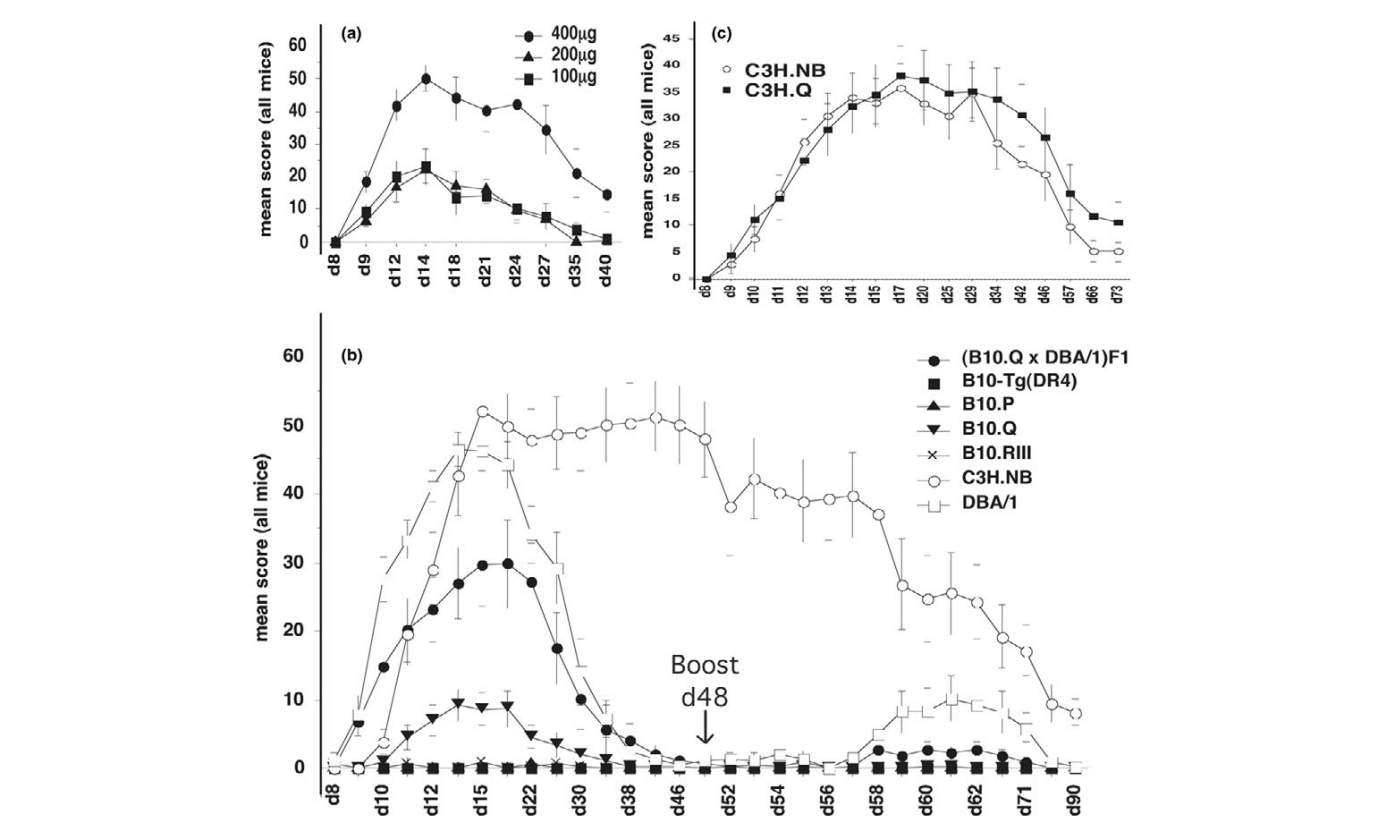
Characterization of G6PI-induced arthritis. Shown are characteristics of glucose-6-phosphate isomerase (G6PI)-induced arthritis in different genetic backgrounds and major histocompatibility complex (MHC) congenics. **(a) **DBA/1 mice were immunized intradermally at the base of the tail with the indicated amounts of G6PI emulsified in complete Freund's adjuvant (CFA) to establish a dynamic immunization protocol allowing for an increase or decrease in disease severity. The course of disease was followed for 40 days after immunization. The graph shows the mean scores for all mice. Disease developed in 8/8 (400 μg/mouse), 9/9 (200 μg/mouse) and 8/9 (100 μg/mouse) mice. **(b) **After establishing the immunization protocol, mice with different MHC haplotypes and genetic backgrounds were immunized with 200 μg G6PI in CFA at the base of the tail. Active arthritis, characterized by redness and oedema, was scored over 90 days after immunization. Blood was drawn at day 40 for antibody analysis and the mice were boosted with human G6PI (50 μg/mouse in incomplete Freund's adjuvant) at day 48. The numbers of mice of each strain evaluated were as follows: (B10.Q × DBA/1)F_1_, *n *= 10; B10-Tg(DR4), *n *= 8; B10.P, *n *= 12; B10.Q, *n *= 26; B10.RIII, *n *= 6; C3H.NB, *n *= 9; and DBA/1, *n *= 10. **(c) **Because the MHC haplotype H-2^p ^on the black background rendered mice resistant to G6PI-induced arthritis, the role of the beta chain of A^p ^was addressed on the highly susceptible C3H background. C3H.NB (H-2^p^; *n *= 21) and C3H.Q (H-2^q^; *n *= 19) mice were immunized intradermally with 200 μg G6PI in CFA and scored for 73 days. At no time point was a significant difference noticed between the two MHC congenic strains. Only a tendency toward more chronic progression could be seen in the C3H.Q mice during the late phase. In all experiments, error bars indicate the standard error of the mean.

Thereafter the disease gradually resolved and no macroscopic signs of arthritis were apparent at day 40 (Fig. 1a), enabling mice to climb under the lids of their cages. The intermediate dose of 200 μg per mouse, which resulted in arthritis in 100%, was selected for further study.

### Pronounced genetic control of chronic arthritis involving both MHC and non-MHC genes

G6PI immunization of different mouse strains resulted in marked differences in arthritis susceptibility and severity. The C3H.NB strain developed arthritis approximately 2 days later than did DBA/1 mice. However, the arthritis in C3H.NB mice was more severe and, most importantly, these mice developed a chronically active inflammation that lasted throughout the observation period of 90 days (Fig. 1b).

**Figure 2:**
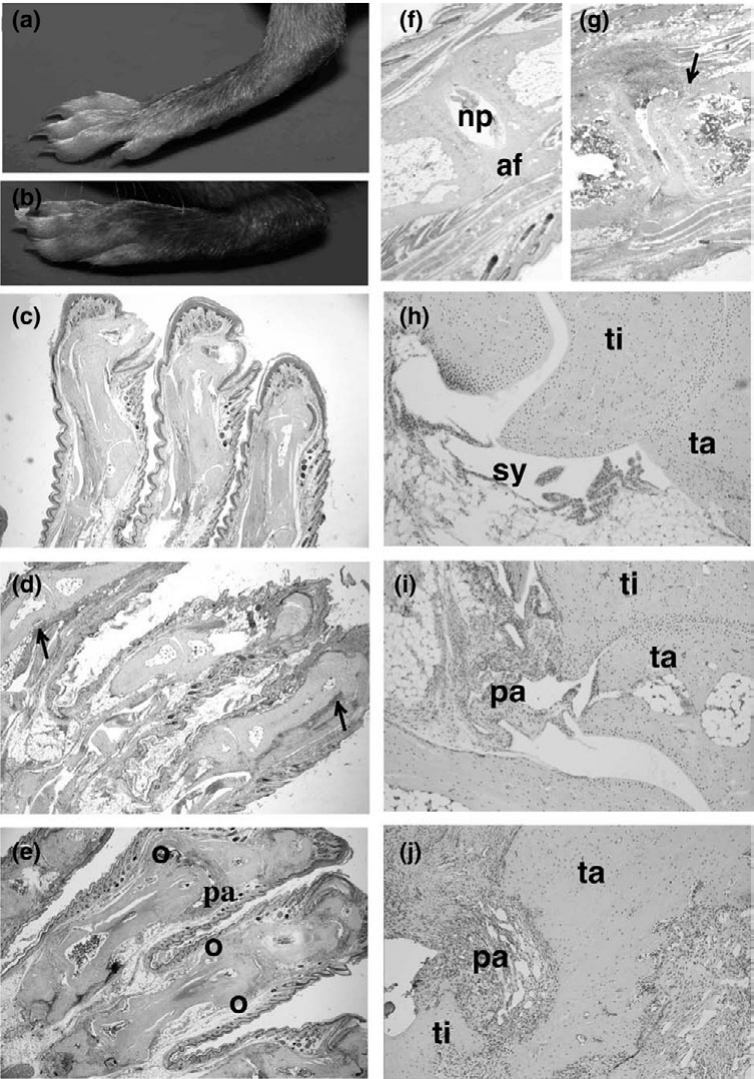
Clinical and histological evaluation of arthritis. Clinical and histological evaluation demonstrates that arthritis induced with 200 μg glucose-6-phosphate isomerase (G6PI) in complete Freund's adjuvant (CFA) leads to chronic destructive arthritis in mice on the C3H background. **(a) **Healthy C3H.Q hind foot. **(b) **A C3H.Q hind foot 90 days after disease induction. The digits are still red and swollen. After day 90 paws were fixated and decalcified for paraffin sectioning. Histopathology demonstrates the destructive character of the GPI-induced arthritis in C3H in comparison with DBA/1 mice. Both mice achieved clinical scores in their hind feet of 15. The C3H mouse developed **(e) **an irreversible destruction of their joints through invasive pannus tissue accompanied by new bone formation, **(j) **destroying the whole architecture of the ankle, whereas DBA/1 mice have relatively intact joints, apart from **(d) **smaller erosions (arrows) and **(i) **hyperplasia. **(c,h) **Healthy control joints. The severity of the disease on the C3H background is also indicated by **(g) **the destruction of intervertebral structures such as the annulus fibrosus, nucleus pulposus and the vertebra themselves by inflammatory cells (arrow). **(f) **Healthy control tail. Staining with haematoxylin and erythrosine; original magnification 25× and 100×. af, annulus fibrosus; np, nucleus pulposus; o, osteophytes; pa, pannus; sy, synovial membrane; ta, talus; ti, tibia.

Histological analysis of joints at 90 days after immunization showed active erosive inflammation (Fig. 2e,j). It should be pointed out that only active inflammatory arthritis with redness and oedema was evaluated for clinical scoring. Even though the oedema declined over time in C3H mice, these animals still had tissue depositions that rendered the joints dysfunctional. There was also massive cell infiltration of the joint space causing erosion and destruction of bone and cartilage, as demonstrated by histology (Fig. 2e,j). The destructive character of this process was striking; for instance, it was even able to dissolve joint cartilage. Simultaneously, new bone formation could be observed, creating large osteophytes. In contrast, DBA/1 mice regained function of many finger joints without major bone remodelling after the inflammation went into remission (Fig. 2d,i), even though this strain is known to be prone to development of osteophytes [27].

The inflammatory process, in contrast to CIA, was not limited to synovial joints but also eroded vertebra and the annulus fibrosus, together with the nucleus pulposus in mice of the C3H background (Fig. 2g). Healthy vertebrae are shown in Fig. 2f. B10.P mice, which share the MHC region with the highly susceptible C3H.NB mice, were resistant to arthritis, showing the strong influence of non-MHC genes. The MHC congenic B10.Q strain, on the other hand, developed significant but mild acute arthritis, whereas another MHC congenic strain – B10.RIII – was also totally resistant to joint destruction. It should be noted that the B10.Q strain used in this study is different from the B10.Q mouse available from Jackson Laboratories, which has an arthritis-protective mutation of the *Tyk2 *gene [28], which explains the earlier reported resistance in these animals to G6PI-induced arthritis [21]. The (B10.Q × DBA/1)F_1 _mice developed arthritis almost as severe as that in DBA/1 mice, showing that part of the genetic contribution from DBA/1 dominates the suppressive B10.Q background genes. Mice expressing the human DR4 (0401) molecule on the B10 background were resistant to arthritis. These observations indicate that the most susceptible MHC haplotype is H2^q^, which is similar to earlier observations in the CIA model [29,30].

Because the highly susceptible C3H.NB strain harboured the less susceptible MHC haplotype (H2^p^), we tested it in comparison with the C3H.Q strain, which is a MHC congenic strain that carries the H2^q ^haplotype. The C3H.Q strain developed slightly more chronic arthritis than the C3H.NB mice, although the difference between the strains in single experiments did not always achieve statistical significance because of the high severity of arthritis in both strains (Fig. 1c).

A booster immunization after resolution of arthritis induced a relapse in most strains (Fig. 1b), but this was milder than the first arthritic episode and started at the exact same time after immunization, suggesting that there is no memory effect from the primary immunization.

### Development of G6PI arthritis is associated with a strong antibody response to G6PI and a weak response to type II collagen

**Figure 3:**
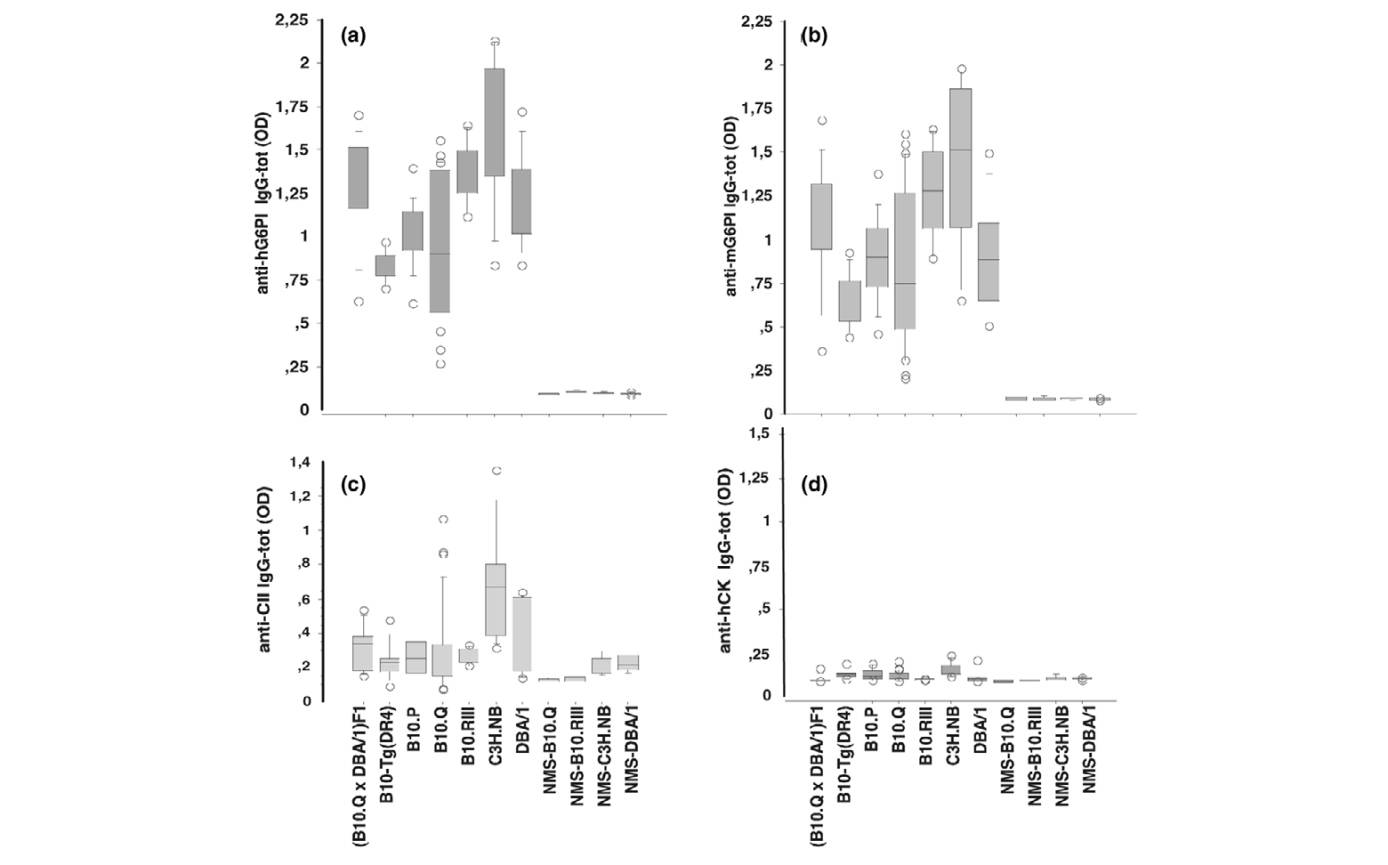
Antibody analysis. Indicated mouse strains were immunized with 200 μg human glucose-6-phosphate isomerase (hG6PI) in complete Freund's adjuvant and bled at day 40 for antibody analysis. ELISA plates were coated with **(a) **10 μg/ml hG6PI, **(b) **mouse G6PI (mG6PI), **(c) **collagen type II (CII), or **(d) **human creatine kinase (hCK). Sera from nonimmunized mice (*n *= 5) of different genetic backgrounds were used as negative controls. The figures show the optical density (OD) value for total IgG responses at a serum dilution of 1:1,000 for hG6PI, mG6PI and hCK (panels a, b and d) and 1:100 for CII (panel c). The results are represented as box plots, indicating the median, the 25th and 75th centiles as boxes, and the 10th and 90th centiles as whiskers. Outliers are indicated as circles.

All arthritis susceptible mouse strains developed a strong antibody response to human G6PI (hG6PI; Fig. 3a). However, the hG6PI specific antibody response did not always correlate with arthritis severity; strong correlation was found only in the C3H.NB strain (Table 1). The anti-G6PI antibody response made use of all IgG isotypes (data not shown). ELISA plates were also coated with recombinant mouse G6PI because human G6PI was used for immunization. The antibody responses were very similar using the two proteins (Fig. 3a,b). To exclude the His-tag as an allogeneic B-cell epitope, we also investigated the antibody response against a His-tag fusion protein of hCK (Fig. 3d) and His-tag labelled recombinant A^q ^as negative controls (data not shown). No significant response for these antigens could be detected. That the response was truly directed against conserved G6PI epitopes was also confirmed by using tissue purified commercial (Sigma-Aldrich Sweden AB, Stockholm, Sweden) rabbit G6PI (data not shown).

Nevertheless, many of the strains used, such as B10.RIII and B10.DR4, did not develop arthritis even though they exhibited strong antibody responses against G6PI, indicating the presence of other protective factors. Interestingly, arthritis susceptible strains developed significant titres to CII (Fig 3c), but again only the C3H.NB mice exhibited a positive correlation between anti-CII antibody response and arthritis severity (Table 1).

### Development of G6PI-induced arthritis is B-cell dependent

**Figure 4:**
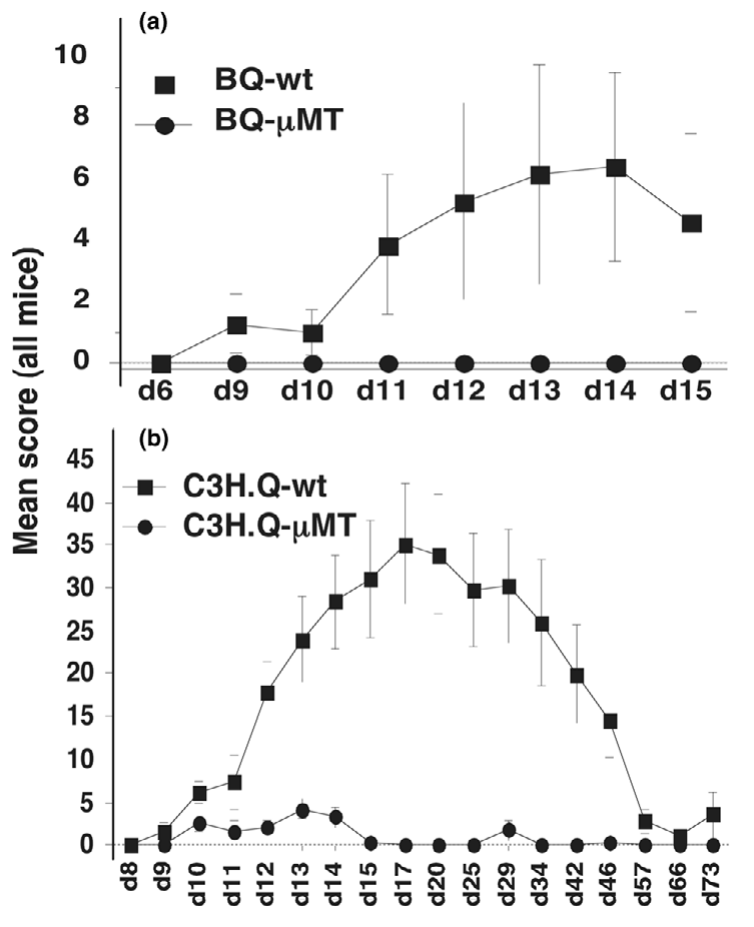
Role of B cells in the pathology of G6PI-induced arthritis. The role of B cells in the pathology of glucose-6-phosphate isomerase (G6PI)-induced arthritis was addressed using B-cell deficient μMT mice on B10.Q and C3H.Q backgrounds. Mice aged between 6 and 7 weeks were immunized with 200 μg G6PI in complete Freund's adjuvant and scored for arthritis. **(a) **Five out of seven B10.Q mice developed arthritis, whereas none of the six B10.Q μMT mice exhibited signs of inflammation. **(b) **Four out of 12 C3H.Q μMT mice exhibited mild oedema for no longer than 48 hours, whereas all nine C3H.Q littermate control mice demonstrated a destructive inflammatory disease course. Error bars indicate the standard error of the mean.

To determine conclusively whether B cells play a critical role in the pathology of G6PI-induced arthritis we used mice with a disrupted IgM gene, which are therefore deficient in mature B cells. No arthritis developed in B-cell-deficient B10.Q mice (Fig. 4a). C3H.Q μMT mice developed only very mild oedema for no longer than 2 days (Fig. 4b), leaving no histological changes (data not shown).

## Discussion

Immunization with G6PI induces arthritis of various degrees of severity and chronicity, depending on the mouse strain. Interestingly, the C3H genetic background permits a chronically active disease course that leads to loss of joint function. This is an important feature of an animal model of RA because the human disease is already chronic when it becomes diagnosed. RA is most likely often preceded by many years of subclinical inflammatory activity. This is not only reflected by raised C-reactive protein levels but also by the production of autoantibodies such as rheumatoid factors and antibodies to citrullinated proteins [31-33]. The chronic disease course of arthritis in C3H.Q and C3H.NB mice, induced with G6PI, will be useful in the analysis of mechanisms of chronicity and as a model to develop new therapeutic protocols.

Interestingly, the C3H background also allows a more severe CIA [16,34]. In addition, in both models, the DBA/1 mouse develops a severe but acute and self-limited type of arthritis. Another striking similarity between the G6PI model and CIA is the association with MHC. In both models the H2^q ^haplotype confers a more severe form of arthritis than does H2^p^. In the CIA model this difference has been shown to be due to the A^q ^molecule, which binds the immunodominant CII_260–270 _glycopeptide with greater affinity than the corresponding A^p ^molecule [35]. It would therefore be of interest to identify the G6PI peptide that binds to A^q ^and to investigate its affinity to the different MHC molecules, in analogy to the CII peptide. However, a difference from CIA is that the H2r haplotype, despite a strong anti-G6PI antibody response, does not confer susceptibility to G6PI-induced arthritis, although in the CIA model the association with H2r is dependent on binding and recognition of peptides other than 260–270 [36]. Another apparent difference is that DR4 (DRB1*0401/DRA) expressing mice are susceptible to CIA [19,37] but not to G6PI-induced arthritis. This is possibly due to a threshold effect, in which the mice developed a strong autoimmune response to G6PI but which, combined with the relative nonpermissive B10 background, did not lead to arthritis. It may not be unexpected that G6PI-induced arthritis is critically dependent on functional B cells, as shown by our findings in B-cell deficient μMT mice on the B10 and the C3H backgrounds. However, it is interesting that a mild transient oedema was observed in B-cell-deficient mice on the highly arthritis susceptible C3H background.

It has been shown in the K/BxN transgenic model that anti-G6PI serum antibodies readily transfer arthritis [4,6]. In the protein-induced G6PI model, this has so far not been demonstrated [21], and the antibody titres in the different strains do not exhibit a convincing correlation with arthritis susceptibility. Thus, high levels of antibodies to G6PI do not always lead to arthritis, indicating that other pathogenic factors play a role. Interestingly, a few mice with severe arthritis developed detectable amounts of antibodies to CII. There is no evidence for a cross-reactivity between G6PI and CII, and the most likely explanation is an activation of an autoimmune response to cartilage-derived CII, as has been seen in pristine-induced arthritis and in various spontaneous arthritides in mice [38-41].

On the C3H background the G6PI model is also useful in investigating ankylosing spondylitis because it generates inflammation of vertebral joints followed by ankylosis after a single round of immunization. Careful serum analysis of patients with different forms of arthritides revealed that G6PI-specific antibodies may be identified not only in severe forms of RA but also in ankylosing spondylitis and Reiter's syndrome [15]. The G6PI-induced arthritis model on the C3H background demonstrates that an initial G6PI immune response is sufficient to induce destructive activity in the spine. Which C3H genetic factors actually contribute to this arthritis pathway remain to be determined, and this needs a careful and cautious analysis of the precise genetic background of any mice used.

Our work over many years with congenic and transgenic mice has made us aware of pitfalls relating to the purity of genetic backgrounds. One should be careful in extrapolating data without having full control over the genetic backgrounds. It is not too surprising that, for instance, the C3H.He mice used by Ji and coworkers [42] did not exhibit high sensitivity for their serum transfer model, as might be suggested by our results. Not only does the C3H.He mouse from Jackson, used by those investigators, has a defect in the Toll-like receptor 4 that renders it unresponsive to lipopolysaccharide stimulation, but also it carries another MHC haplotype (H2-K) compared with our congenic mice. Furthermore, it is likely that the different MHC congenic inbred strains have accumulated mutations over the years, as well as carrying several contaminating fragments due to incomplete backcrossing. Bearing these problems in mind, we backcrossed our C3H.Q mice for several generations to C3H.NB to be sure that we compared the MHC effect only. Therefore, it will be of great interest in future investigations to use a panel of highly controlled congenic mice to identify chronicity factors in C3H mice.

In an examination of the IgG isotypes active in human Reiter's syndrome, they appeared to be predominantly of the T-helper-2-like isotype IgG_4_, equivalent to IgG_1 _in K/BxN mice [15]. It will be interesting to investigate whether a T-helper-2 driven immune response is responsible for the chronic severity and spine involvement with the C3H background.

Taken together, there are several similarities but also differences between G6PI-induced arthritis and CIA. Most strikingly, the genetic control in the two models allows only acute arthritis in DBA/1 mice but a more chronic relapsing form in mice of the C3H background. In the CIA model the B10 background allows chronic development of arthritis [43]. Interestingly, both models appear to follow a central pathogenic pathway that involves B-cell autoreactivity and arthritogenic antibodies. Experiments using antibodies to CII and G6PI over the years have shown extensive similarities [6,8,42,44-49].

The most obvious difference between the two models is the tissue distribution of the autoantigen. G6PI is systemically distributed in the body because it is expressed intracellular in all cells as an enzyme of glycolysis and can furthermore be secreted. CII is also widely expressed during foetal development (for review see Holmdahl and coworkers [50]). In the adult CII expression is more restricted, mainly to cartilage (e.g. in diarthrodial joints, larynx, spine and sternum). It is also expressed in the vitreous body of the eye [50]. G6PI-induced arthritis developed much earlier after immunization than did CIA and with a stronger oedematous appearance, which could extend into the knees, although no prominent histological changes were observed in DBA/1 mice. Thus, in both CIA and G6PI-induced arthritis the tissue distribution of the autoantigen could not account for the specificity of the inflammatory disease. In fact, models with unknown autoantigens, like induction of arthritis with the alkane pristane, are also specific in that the resulting inflammation only affects joints [51,52].

One important question to address is where the immune system detects the autoantigen. The antigen might be differently expressed in various tissues and be processed differently depending on the kind of antigen-presenting cell. Another issue to address is the role played by synovial tissue in diarthrodial joints because these joints are predominantly affected. The cartilage surface may be of importance for triggering antibody-mediated inflammation, as shown for both anti-CII and anti-G6PI antibodies. Taken together, we believe that G6PI-induced arthritis is a very useful model for studies of RA and it may represent a unique pathway, in particular with respect to its autoantigen specificity and chronicity.

## Conclusion

This study showed that G6PI-induced arthritis can be converted into a chronic inflammatory arthritis model by using the C3H genetic background. Mice of the C3H background also develop arthritis in their vertebra, supporting a role for G6PI reactivity in ankylosing spondylitis.

We conclude that G6PI has the ability to induce a chronic from of arthritis, which is MHC associated and B-cell dependent. Thus, there is a striking similarity between G6PI-induced arthritis and the CIA model. Genetic factors determining chronicity – a hallmark of RA – will be addressed using this model in future experiments.

## Abbreviations

CIA = collagen-induced arthritis; CII = collagen type II; ELISA = enzyme-linked immunosorbent assay; G6PI = glucose-6-phosphate isomerase; hCK = human creatine kinase; MHC = major histocompatibility complex; RA = rheumatoid arthritis; rCII = rat collagen type II.

## Competing interests

The author(s) declare that they have no competing interests.

## Authors' contributions

RB performed the experiments, and was involved in designing the study and in writing the manuscript under the guidance of RH. DS and TK produced the recombinant proteins (hG6PI, mG6Pi and hCK) and were involved in designing the study and critically read the manuscript.

## Figures and Tables

**Table 1:** Correlation between specific IgG-total and accumulative score

Strain	Anti-hG6PI versus score		Anti-CII versus score		*n*
		
	Rho	*P *value	Rho	*P *value	
C3H.NB	0.917*	0.009*	0.793*	0.026*	9
B10.Q	0.402*	0.046*	-0.094	0.614	26
(B10.Q × DBA1)F_1_	0.018	0.956	0.139	0.67	10
DBA/1	0.317	0.370	0.033	0.924	10

To investigate correlations between arthritis severity and antibody production the Spearman correlation test was applied. Mice of indicated strains were immunized with 200 μg glucose-6-phosphate isomerase (G6PI) in complete Freund's adjuvant. Blood was drawn at day 40 and analyzed by ELISA for anti-hG6PI and anti-CII total IgG responses, as shown in Fig. 1. The accumulative arthritis score until day 40 was tested for correlation with antibody production using the Spearman rank correlation test. A rho value close to 1 indicates correlation of high ranks for IgG with high ranks for arthritis scores; 0 indicates that there is no correlation between values; and a number close to -1 indicates that high ranks for one variable correlate with low ranks for the other. *Significant positive correlations.

## References

[B1] Sun YJ, Chou CC, Chen WS, Wu RT, Meng M, Hsiao CD (1999). The crystal structure of a multifunctional protein: phosphoglucose isomerase/autocrine motility factor/neuroleukin. Proc Natl Acad Sci USA.

[B2] Chou CC, Sun YJ, Meng M, Hsiao CD (2000). The crystal structure of phosphoglucose isomerase/autocrine motility factor/neuroleukin complexed with its carbohydrate phosphate inhibitors suggests its substrate/receptor recognition. J Biol Chem.

[B3] Korganow AS, Ji H, Mangialaio S, Duchatelle V, Pelanda R, Martin T, Degott C, Kikutani H, Rajewsky K, Pasquali L (1999). From systemic T cell self reactivity to organ-specific autoimmune disease via immunoglobulins. Immunity.

[B4] Matsumoto I, Staub A, Benoist C, Mathis D (1999). Arthritis provoked by linked T and B cell recognition of a glycolytic enzyme. Science.

[B5] Matsumoto I, Maccioni M, Lee DM, Maurice M, Simmons B, Brenner M, Mathis D, Benoist C (2002). How antibodies to a ubiquitous cytoplasmic enzyme may provoke joint-specific autoimmune disease. Nat Immunol.

[B6] Maccioni M, Zeder-Lutz G, Huang H, Ebel C, Gerber P, Hergueux J, Marchal P, Duchatelle V, Degott C, van egenmortel M (2002). Arthritogenic monoclonal antibodies from K/BxN mice. J Exp Med.

[B7] Stuart JM, Dixon FJ (1983). Serum transfer of collagen induced arthritis in mice. J Exp Med.

[B8] Holmdahl R, Rubin K, Klareskog L, Larsson E, Wigzell H (1986). Characterization of the antibody response in mice with type II collagen-induced arthritis, using monoclonal anti-type II collagen antibodies. Arthritis Rheum.

[B9] Mandik-Nayak L, Wipke BT, Shih FF, Unanue ER, Allen PM (2002). Despite ubiquitous autoantigen expression, arthritogenic autoantibody response initiates in the local lymph node. Proc Natl Acad Sci USA.

[B10] Schaller M, Burton DR, Ditzel HJ (2001). Autoantibodies to GPI in rheumatoid arthritis: linkage between an animal model and human disease. Nat Immunol.

[B11] Schubert D, Schmidt M, Zaiss D, Jungblut PR, Kamradt T (2002). Autoantibodies to GPI and creatine kinase in RA. Nat Immunol.

[B12] Kassahn D, Kolb C, Solomon S, Bochtler P, Illges H (2002). Few human autoimmune sera detect GPI. Nat Immunol.

[B13] Matsumoto I, Lee DM, Goldbach-Mansky R, Sumida T, Hitchon CA, Schur PH, Anderson RJ, Coblyn JS, Weinblatt ME, Brenner M (2003). Low prevalence of antibodies to glucose-6-phosphate isomerase in patients with rheumatoid arthritis and a spectrum of other chronic autoimmune disorders. Arthritis Rheum.

[B14] van Gaalen FA, Toes RE, Ditzel HJ, Schaller M, Breedveld FC, Verweij CL, Huizinga TW (2004). Association of autoantibodies to glucose-6-phosphate isomerase with extraarticular complications in rheumatoid arthritis. Arthritis Rheum.

[B15] Schaller M, Stohl W, Tan SM, Benoit VM, Hilbert DM, Ditzel HJ (2005). Raised levels of anti-glucose-6-phosphate isomerase IgG in serum and synovial fluid from patients with inflammatory arthritis. Ann Rheum Dis.

[B16] Holmdahl R, Andersson M, Goldschmidt TJ, Gustafsson K, Jansson L, Mo JA (1990). Type II collagen autoimmunity in animals and provocations leading to arthritis. Immunol Rev.

[B17] Kjellen P, Jansson L, Vestberg M, Andersson AA, Mattsson R, Holmdahl R (2001). The H2-Ab gene influences the severity of experimental allergic encephalomyelitis induced by proteolipoprotein peptide 103–116. J Neuroimmunol.

[B18] Svensson L, Jirholt J, Holmdahl R, Jansson L (1998). B cell-deficient mice do not develop type II collagen-induced arthritis (CIA). Clin Exp Immunol.

[B19] Andersson EC, Hansen BE, Jacobsen H, Madsen LS, Andersen CB, Engberg J, Rothbard JB, Sönderstrup-McDevitt G, Malmström V, Holmdahl R (1998). Definition of MHC and T cell receptor contacts in the HLA-DR4 restricted immunodominant epitope in type II collagen and characterization of collagen-induced arthritis in HLA-DR4 and human CD4 transgenic mice. Proc Natl Acad Sci USA.

[B20] Bäcklund J, Carlsen S, Höger T, Holm B, Fugger L, Kihlberg J, Burkhardt H, Holmdahl R (2002). Predominant selection of T cells specific for glycosylated collagen type II peptide (263–270) in humanized transgenic mice and in rheumatoid arthritis. Proc Natl Acad Sci USA.

[B21] Schubert D, Maier B, Morawietz L, Krenn V, Kamradt T (2004). Immunization with glucose-6-phosphate isomerase induces T cell-dependent peripheral polyarthritis in genetically unaltered mice. J Immunol.

[B22] Smith BD, Martin GR, Dorfman A, Swarm R (1975). Nature of the collagen synthesized by a transplanted chondrosarcoma. Arch Biochem Biophys.

[B23] Andersson M, Holmdahl R (1990). Analysis of type II collagen reactive T cells in the mouse. I. Different regulation of autoreactive versus non-autoreactive anti-type II collagen T cells in the DBA/1 mouse. Eur J Immunol.

[B24] Holmdahl R (1998). Animal models for rheumatoid arthritis useful for drug screening and evaluation. Curr Res Rheum Arthr.

[B25] Holmdahl R, Jansson L, Larsson E, Rubin K, Klareskog L (1986). Homologous type II collagen induces chronic and progressive arthritis in mice. Arthritis Rheum.

[B26] Holmdahl R, Jonsson R, Larsson P, Klareskog L (1988). Early appearence of activated CD4 positive T lymphocytes and Ia-expressing cells in joints of DBA/1 mice immunized with type II collagen. Lab Invest.

[B27] Corthay A, Hansson AS, Holmdahl R (2000). T lymphocytes are not required for the spontaneous development of entheseal ossification leading to marginal ankylosis in the DBA/1 mouse. Arthritis Rheum.

[B28] Shaw MH, Boyartchuk V, Wong S, Karaghiosoff M, Ragimbeau J, Pellegrini S, Muller M, Dietrich WF, Yap GS (2003). A natural mutation in the Tyk2 pseudokinase domain underlies altered susceptibility of B10.Q/J mice to infection and autoimmunity. Proc Natl Acad Sci USA.

[B29] Wooley PH, Luthra HS, Stuart JM, David CS (1981). Type II collagen induced arthritis in mice. I. Major histocompatibility complex (I-region) linkage and antibody correlates. J Exp Med.

[B30] Brunsberg U, Gustafsson K, Jansson L, Michaëlsson E, Ährlund-Richter L, Pettersson S, Mattsson R, Holmdahl R (1994). Expression of a transgenic class II Ab gene confers susceptibility to collagen-induced arthritis. Eur J Immunol.

[B31] Aho K, Palosuo T, Raunio V, Puska P, Aromaa A, Salonen JT (1985). When does rheumatoid disease start?. Arthritis Rheum.

[B32] Rantapaa-Dahlqvist S, de Jong BA, Berglin E, Hallmans G, Wadell G, Stenlund H, Sundin U, van Venrooij WJ (2003). Antibodies against cyclic citrullinated peptide and IgA rheumatoid factor predict the development of rheumatoid arthritis. Arthritis Rheum.

[B33] van Gaalen FA, Linn-Rasker SP, van Venrooij WJ, de Jong BA, Breedveld FC, Verweij CL, Toes RE, Huizinga TW (2004). Autoantibodies to cyclic citrullinated peptides predict progression to rheumatoid arthritis in patients with undifferentiated arthritis: a prospective cohort study. Arthritis Rheum.

[B34] Bäcklund J, Nandakumar KS, Bockermann R, Mori L, Holmdahl R (2003). Genetic control of tolerance to type II collagen and development of arthritis in an autologous collagen-induced arthritis model. J Immunol.

[B35] Kjellén P, Brunsberg U, Broddefalk J, Hansen B, Vestberg M, Ivarsson I, Engström Å, Svejgaard A, Kihlberg J, Fugger L (1998). The structural basis of MHC control of collagen-induced arthritis; binding of the immunodominant type II collagen 256–270 glycopeptide to H-2Aq and H-2Ap molecules. Eur J Immunol.

[B36] Myers LK, Miyahara H, Terato K, Seyer JM, Stuart JM, Kang AH (1995). Collagen-induced arthritis in B10.RIII mice (H-2r): identification of an arthritogenic T-cell determinant. Immunology.

[B37] Rosloniec EF, Brand DD, Myers LK, Esaki Y, Whittington KB, Zaller DM, Woods A, Stuart JM, Kang AH (1998). Induction of autoimmune arthritis in HLA-DR4 (DRB1*0401) transgenic mice by immunization with human and bovine type II collagen. J Immunol.

[B38] Wooley PH, Seibold JR, Whalen JD, Chapdelaine JM (1989). Pristane-induced arthritis. The immunologic and genetic features of an experimental murine model of autoimmune disease. Arthritis Rheum.

[B39] Hultqvist M, Olofsson P, Holmberg J, Bäckström BT, Tordsson J, Holmdahl R (2004). Enhanced autoimmunity, arthritis, and encephalomyelitis in mice with a reduced oxidative burst due to a mutation in the Ncf1 gene. Proc Natl Acad Sci USA.

[B40] Sakaguchi N, Takahashi T, Hata H, Nomura T, Tagami T, Yamazaki S, Sakihama T, Matsutani T, Negishi I, Nakatsuru S, Sakaguchi S (2003). Altered thymic T-cell selection due to a mutation of the ZAP-70 gene causes autoimmune arthritis in mice. Nature.

[B41] Kotani M, Tagawa Y, Iwakura Y (1999). Involvement of autoimmunity against type II collagen in the development of arthritis in mice transgenic for the human T cell leukemia virus type I tax gene. Eur J Immunol.

[B42] Ji H, Gauguier D, Ohmura K, Gonzalez A, Duchatelle V, Danoy P, Garchon HJ, Degott C, Lathrop M, Benoist C (2001). Genetic influences on the end-stage effector phase of arthritis. J Exp Med.

[B43] Svensson L, Nandakumar KS, Johansson A, Jansson L, Holmdahl R (2002). IL-4-deficient mice develop less acute but more chronic relapsing collagen-induced arthritis. Eur J Immunol.

[B44] Ji H, Ohmura K, Mahmood U, Lee DM, Hofhuis FM, Boackle SA, Takahashi K, Holers VM, Walport M, Gerard C (2002). Arthritis critically dependent on innate immune system players. Immunity.

[B45] Stuart JM, Tomoda K, Yoo TJ, Townes AS, Kang AH (1983). Serum transfer of collagen-induced arthritis. II. Identification and localization of autoantibody to type II collagen in donor and recipient rats. Arthritis Rheum.

[B46] Terato K, Hasty KA, Reife RA, Cremer MA, Kang AH, Stuart JM (1992). Induction of arthritis with monoclonal antibodies to collagen. J Immunol.

[B47] Hietala MA, Nandakumar KS, Persson L, Fahlen S, Holmdahl R, Pekna M (2004). Complement activation by both classical and alternative pathways is critical for the effector phase of arthritis. Eur J Immunol.

[B48] Nandakumar KS, Svensson L, Holmdahl R (2003). Collagen type II specific monoclonal antibody induced arthritis (CAIA) in mice. Description of the disease and the influence of age, sex, and genes. Am J Pathol.

[B49] Nandakumar KS, Bäcklund J, Vestberg M, Holmdahl R (2004). Collagen type II (CII)-specific antibodies induce arthritis in the absence of T or B cells but the arthritis progression is enhanced by CII-reactive T cells. Arthritis Res Ther.

[B50] Holmdahl R, Malmström V, Vuorio E (1993). Autoimmune recognition of cartilage collagens. Ann Med.

[B51] Vingsbo C, Sahlstrand P, Brun JG, Jonsson R, Saxne T, Holmdahl R (1996). Pristane-induced arthritis in rats: a new model for rheumatoid arthritis with a chronic disease course influenced by both major histocompatibility complex and non-major histocompatibility complex genes. Am J Pathol.

[B52] Hansson AS, Lu S, Holmdahl R (2002). Extra-articular cartilage affected in collagen-induced, but not pristane-induced, arthritis models. Clin Exp Immunol.

